# Dysfunction of the blood-brain barrier in postoperative delirium patients, referring to the axonal damage biomarker phosphorylated neurofilament heavy subunit

**DOI:** 10.1371/journal.pone.0222721

**Published:** 2019-10-01

**Authors:** Kazuhito Mietani, Masahiko Sumitani, Toru Ogata, Nobutake Shimojo, Reo Inoue, Hiroaki Abe, Gaku Kawamura, Yoshitsugu Yamada

**Affiliations:** 1 Department of Anesthesiology and Pain Relief Center, The University of Tokyo Hospital, Tokyo, Japan; 2 Department of Pain and Palliative Medicine, The University of Tokyo Hospital, Tokyo, Japan; 3 Department of Rehabilitation for the Movement Functions, Research Institute, National Rehabilitation Center for Persons with Disabilities, Saitama, Japan; 4 Department of Emergency and Critical Care Medicine, Tsukuba University Hospital, Ibaraki, Japan; Tokyo Metropolitan Institute of Medical Science, JAPAN

## Abstract

**Background:**

Delirium is the most common postoperative complication of the central nervous system (CNS) that can trigger long-term cognitive impairment. Its underlying mechanism is not fully understood, but the dysfunction of the blood-brain barrier (BBB) has been implicated. The serum levels of the axonal damage biomarker, phosphorylated neurofilament heavy subunit (pNF-H) increase in moderate to severe delirium patients, indicating that postoperative delirium can induce irreversible CNS damage. Here, we investigated the relationship among postoperative delirium, CNS damage and BBB dysfunction, using pNF-H as reference.

**Methods:**

Blood samples were collected from 117 patients within 3 postoperative days. These patients were clinically diagnosed with postoperative delirium using the Confusion Assessment Method for the Intensive Care Unit. We measured intercellular adhesion molecule-1, platelet and endothelial cell adhesion molecule-1, vascular cell adhesion molecule-1, E-selectin, and P-selectin as biomarkers for BBB disruption, pro-inflammatory cytokines (tumor necrosis factor-alpha, interleukin-1 beta, interleukin-6), and pNF-H. We conducted logistic regression analysis including all participants to identify independent biomarkers contributing to serum pNF-H detection. Next, by multiple regression analysis with a stepwise method we sought to determine which biomarkers influence serum pNF-H levels, in pNF-H positive patients.

**Results:**

Of the 117 subjects, 41 were clinically diagnosed with postoperative delirium, and 30 were positive for serum pNF-H. Sensitivity and specificity of serum pNF-H detection in the patients with postoperative delirium were 56% and 90%, respectively. P-selectin was the only independent variable to associate with pNF-H detection (*P* < 0.0001) in all 117 patients. In pNF-H positive patients, only PECAM-1 was associated with serum pNF-H levels (*P* = 0.02).

**Conclusions:**

Serum pNF-H could be an objective delirium biomarker, superior to conventional tools in clinical settings. In reference to pNF-H, P-selectin may be involved in the development of delirium-related CNS damage and PECAM-1 may contribute to the progression of delirium- related CNS damage.

## Introduction

Delirium is the most common neurological complication of the central nervous system (CNS) during postoperative periods. The incidence of delirium can affect postoperative recovery and prognosis. For example, it is associated with increased mortality and morbidity [1], prolonged stays in the intensive care unit (ICU) [2], and higher hospital costs [3,4]. However the diagnosis of delirium by ICU physicians has been reported poor (sensitivity = 29%) [5]. Further, its adverse effects can be sustained and lead to long-term cognitive impairment and brain atrophy even in patients who recover from postoperative delirium [6]. Mechanism-based diagnostics and severity measures of postoperative delirium are urgently required in clinical settings.

Based on the previous notion that the incidence of postoperative delirium is linked with brain atrophy [7], we measured the serum levels of phosphorylated neurofilament heavy subunit (pNF-H), a major structural protein of the CNS axons, in patients with postoperative delirium [8]. Consequently, 65% of the patients with delirium were positive for serum pNF-H. Because pNF-H is a structural protein of CNS axons, it is not detectable in the blood under healthy conditions. Therefore, the detection of pNF-H indicates neural tissue damage in delirium patients. Furthermore, the serum levels of pNF-H were correlated with the clinical severity of delirium [8]. Considering that the severity of spinal cord injury was associated with serum pNF-H levels [9], our previous findings suggested a potential application of pNF-H as a biomarker of neural tissue damage in delirium [10].

One mechanism of delirium has been suggested to implicate inflammation of the blood-brain barrier (BBB) [11], because inflammatory conditions such as sepsis and wound infection are major causes of delirium [12,13]. Indeed, there are studies demonstrating that elevated levels of plasma markers of BBB disruption (e.g., intercellular adhesion molecule-1 [ICAM-1], platelet and endothelial cell adhesion molecule-1 [PECAM-1], vascular cell adhesion molecule-1 [VCAM-1], E-selectin, and P-selectin) [14,15] or pro-inflammatory cytokines (e.g., interleukin [IL]-6, IL-1 beta [IL-1β], tumor necrosis factor-alfa [TNF-α]) [11,16,17] are associated with delirium in serious diseases. In inflammatory states, the endothelial activation leads to microcirculatory blood flow abnormalities and leukocyte adhesion on the BBB. This may contribute to increased BBB permeability and finally to neural tissue injury given that the BBB is composed of an endothelial layer with tight junctions and astrocyte end-feet processes [14]. In the present study, we focused on the potential application of serum pNF-H as an objective biomarker of the development and severity of delirium, and we elucidated an relationship between delirium and BBB dysfunction.

## Materials and methods

### Ethics

This study was conducted at the University of Tokyo Hospital, Saitama Red Cross Hospital and Tsukuba University Hospital, from July 2013 to June 2016. The local ethics committee of each institution approved the trial protocol and written informed consent was obtained from each patient before participating in the study. This study was registered in the University Medical Information Network (UMIN trial ID: UMIN000010329).

### Study population

Patients scheduled to undergo non-cardiac surgery for cancer under general anesthesia (irrespective of the affected organ) were included in this study. Among these, patients with less than 4 in the American Society of Anesthesiologists (ASA) physical classification [18] were enrolled. Patients with slight, or more, clinically relevant cognitive dysfunction before surgery and those diagnosed with cerebrospinal lesions were excluded. Patients taking tranquilizers before surgery that could have a prophylactic effect on delirium [19] were also excluded. Further, we excluded patients who required neurosurgery for lesions located in the brain and spinal cord, or cardiothoracic surgery with a cardiopulmonary bypass, because such procedures can lead to nerve destruction and ischemia in the brain and spinal cord, and consequently increase serum pNF-H levels.

### Patient assessment

Within 1 week after surgery, the patients were assessed for the following delirium-associated symptoms by the attending nurses, at least three times a day during regular ward rounds: (i) verbal or behavioral manifestation of not being oriented in time or place, or misperceiving persons in the environment; (ii) insomnia, daytime somnolence, and day-night reversal; (iii) restlessness or agitation, irritability, excitement, and confusion; (iv) inappropriate behavior; (v) inappropriate communication; (vi) tendency to see or hear things that do not exist and distortions of visual objects. Suspected postoperative delirium was confirmed by the investigators according to the Confusion Assessment Method for the Intensive Care Unit (CAM-ICU) [20]. Blood samples were collected on postoperative day 3. We measured serum pNF-H levels using a commercially available enzyme-linked immunosorbent assay (ELISA) kit (Human Phosphorylated Neurofilament H ELISA; BioVendor, Modrice, Czech Republic) according to the manufacturer’s protocol. Serum pNF-H levels higher than 70.5 pg/mL were considered positive [21]. Further, delirium is likely associated with elevated plasma markers of endothelial activation and BBB dysfunction, such as ICAM-1, PECAM-1, VCAM-1, E-selectin, P-selectin [22,23]. Therefore, we sought to measure their serum levels. Moreover, surgical stimulus triggers the release of pro-inflammatory mediators and cytokines including IL-6, IL-1β, TNF-α, all of which have been reportedly associated with the development of delirium [11,16,17]. These pro-inflammatory cytokines can lead to endothelial dysfunction and dysregulate the permeability of the BBB [16]. Based on the above information, we also measured the serum levels of these three pro-inflammatory cytokines. All endothelial markers and pro-inflammatory cytokines were measured using a commercially available ELISA kit (Procarta Immunoassay kit, human by request; Panomics, Inc, CA).

### Statistical analysis

We previously revealed that 65% of patients with clinically-relevant delirium are positive for serum pNF-H, and the serum levels of pNF-H are significantly linked with delirium severity [8]. In the present study, we calculated the sensitivity and specificity of serum pNF-H detection for the diagnosis of delirium. Our study aimed at two additional targets; first, we aimed to identify independent biomarkers contributing to serum pNF-H positivity, which was used as a proxy of postoperative delirium with anatomical neuronal damage. We conducted a logistic regression analysis on participants both with dependent variable and without postoperative delirium. We set BBB injury biomarkers and pro-inflammatory cytokines as independent variables, and serum pNF-H positivity as the dependent variable. Second, we aimed to determine whether these biomarkers influence serum pNF-H levels as a biomarker for the estimation of clinical severity of delirium in the patients with serum pNF-H detection. We conducted multiple regression analysis with a stepwise method using BBB injury biomarkers and pro-inflammatory cytokines as independent variables and serum pNF-H levels as the dependent variable. The differences in baseline characteristics were tested using chi-square tests. All analyses were performed using the SPSS statistical software, version 22 [IBM Corp, Armonk, NY]. *P* values of (or lower than) 0.05 were considered statistically significant.

## Results

A total of 117 patients undergoing elective surgery under general anesthesia provided written informed consent for their participation in the present study. The baseline characteristics of the 117 patients, either positive or negative for serum pNF-H, are presented in Table 1. There were no significant differences in the clinical backgrounds among patients, except for age [Table 1]. Of the 117 patients, 41 were clinically diagnosed with delirium during postoperative periods, and in 23 of these patients increased serum pNF-H levels were detected. In addition, 7 patients without clinically-diagnosed delirium demonstrated pNF-H positive sera. The sensitivity and specificity of serum pNF-H detection for the patients with postoperative delirium were 56% and 90%, respectively.

**Table 1:** Characteristics of the study population.

	PNF-H POSITIVE	PNF-H NEGATIVE	*P* VALUE
Number	30	87	
Delirium +/-	23/7	18/69	< 0.0001^#^
Age (yr)	76.9 ± 5.24	66.2 ± 12.01	< 0.0001^#^
Sex, male (%)	18 (60.0%)	46 (52.87%)	0.53
Body mass index (kg/m^2^)	23.2 ± 6.75	23.0 ± 5.2	0.85

^#^, *P* value < 0.05.

To identify factors contributing to the development of delirium with neuronal damage during postoperative periods we performed logistic regression analysis including all 117 patients. Our results revealed that, among the examined BBB biomarkers and pro-inflammatory cytokines, P-selectin was the only independent variable that associated with serum pNF-H detection (*P* < 0.0001; Table 2). Furthermore, among the 30 patients that were positive for serum pNF-H, only PECAM-1 was associated with increased serum pNF-H levels, as identified by multiple regression analysis with a stepwise method (*P* = 0.02; Table 3).

**Table 2:** Results of logistic regression analysis on all participants to identify independent biomarkers contributing to serum pNF-H detection during postoperative periods.

	SERUM LEVELS (PG/ML) (MEAN ± SD)	B	SEM	WALD	DEGREE OF FREEDOM	*P* VALUE
Constant	-	-2.742	1.093	6.294	1	0.01
ICAM-1	110666.7 ± 238761.2	0.000	0.000	1.048	1	0.31
PECAM-1	12680.6 ± 3865.5	0.000	0.000	0.229	1	0.63
VCAM-1	523359.9 ± 596216.9	0.000	0.000	2.016	1	0.16
E-selectin	44155.2 ± 23810.7	0.000	0.000	1.131	1	0.29
P-selectin	99582.3 ± 67763.2	0.000	0.000	12.604	1	0.0004^#^
TNF-α	4.1 ± 15.4	-0.117	0.103	1.301	1	0.25
IL-1β	3.6 ± 7.2	-0.038	-0.038	0.152	1	0.70
IL-6	167.5 ± 393.1	-0.001	-0.001	0.268	1	0.60

ICAM-1, intercellular adhesion molecule-1; PECAM-1, platelet and endothelial cell adhesion molecule-1; VCAM-1, vascular cell adhesion molecule-1; IL-6, interleukin-6; IL-1β, interleukin-1 beta; TNF-α, tumor necrosis factor-alfa [TNF-α];

^#^, *P* value < 0.05.

**Table 3:** Results of the stepwise multiple regression analysis performed to determine whether biomarkers correlate to pNF-H levels in patients with serum pNF-H.

	SERUM LEVELS (PG/ML) (MEAN ± SD)	B	SE	STANDARDIZED REGRESSION COEFFICIENT	T VALUE	*P* VALUE
Constant	-	-276.507	1.093	-	-0.824	0.42
PECAM-1	13441.5 ± 4036.5	5.932	0.024	0.425	2.481	0.019^#^
ICAM-1	161527.3 ± 325449.8	-	-	-0.136	-0.774	0.45
VCAM-1	644607.8 ± 797160.3	-	-	-0.102	-0.571	0.57
E-selectin	36129.4 ± 14830.6	-	-	-0.218	-1.185	0.25
P-selectin	159320.0 ± 70313.1	-	-	0.095	0.544	0.59
TNF-α	2.1 ± 2.8	-	-	0.167	0.970	0.34
IL-1β	1.9 ± 3.8	-	-	0.107	0.604	0.55
IL-6	54.3 ± 88.4	-	-	-0.047	-0.269	0.79

ICAM-1, intercellular adhesion molecule-1; PECAM-1, platelet and endothelial cell adhesion molecule-1; VCAM-1, vascular cell adhesion molecule-1; IL-6, interleukin-6; IL-1β, interleukin-1 beta; TNF-α, tumor necrosis factor-alfa [TNF-α];

^#^, *P* value < 0.05.

## Discussion

Delirium is the most common neurological postoperative complication of the CNS. A serum biomarker of CNS axonal damage, pNF-H, demonstrated relatively low sensitivity but high specificity for clinically-relevant postoperative delirium. Focusing on the known relationship between delirium and BBB dysfunction, we revealed that P-selectin contributes to the development of delirium-related CNS damage. Moreover, serum pNF-H levels, a potential biomarker for the estimation of clinical severity of delirium, were associated with PECAM-1. All measured pro-inflammatory cytokines were not directly associated with either the development or severity of postoperative delirium-related CNS damage, using serum pNF-H levels as a proxy.

In several CNS pathological conditions, immune cells are considered to play an important role in CNS anatomical damage. There are several steps in the process by which immune cells invade into the CNS through the BBB and finally exert CNS cytotoxicity. Immune cells, such as neutrophils, lymphocytes, and monocytes cannot normally migrate into the CNS because of their size, and the function and integrity of the BBB [11]. During systemic inflammation, for example in cases of surgical invasion and sepsis, the expression of cell adhesion molecules and pro-inflammatory cytokines increases and subsequently the tight junctions connecting the vascular endothelial cells of the BBB are disrupted, so that immune cells and pro-inflammatory molecules can cross the BBB [14]. The process by which immune cells pass through the BBB and invade the CNS includes four sequential steps: the immune cells (1) capture the vascular endothelium, (2) roll along the vascular endothelium while decelerating, (3) immobilize (firm adhesion to the endothelium), and (4) pass through the vascular endothelial cells (transmigration) [24,25]. P-selectin is one of the cell adhesion molecules involved in the initial “capture” step [26]. In the present study, we demonstrated that P-selectin is associated with serum pNF-H detection. Considering this finding, P-selectin may be a critical player in the development of postoperative delirium-related CNS damage, and could also be considered as a potential therapeutic target for preventing postoperative delirium-related CNS damage onset. Further, we found that PECAM-1 is associated with increased pNF-H serum levels which is considered as a surrogate biomarker for the estimation of clinical severity of delirium [8]. PECAM-1 is one of the cell adhesion molecules that participate in the final “transmigration” step. Through this step, immune cells migrate into the CNS and finally exert their neurotoxic effect [27]. Therefore, PECAM-1 might play a crucial role in the progression of the anatomical CNS damage, observed subsequently to delirium onset. Moreover, PECAM-1 could be considered as a potential therapeutic target for protecting the CNS from the cytotoxicity exerted by the immune cells and preventing the sustained cognitive impairment observed in conjunction with brain atrophy, even later than 6 months after surgery [28,29]. Thus, our present findings suggest two candidate cell adhesion molecules of the BBB that may be responsible for the development and severity of postoperative delirium-related CNS damage, correlating with serum detection of the axonal damage biomarker, pNF-H.

The sensitivity and specificity of serum pNF-H detection in the patients with postoperative delirium were 56% and 90%, respectively. The consequences of delirium include prolonged hospital stays and higher hospital costs, higher mortality, and morbidity. Therefore, the diagnosis of delirium should become more efficient. At present, CAM-ICU is one of the most widely used diagnostic tools by the ICU physicians [30]. However, CAM-ICU requires that the physicians have solid experience and technical expertise. Hence, the diagnosis of delirium is difficult in practice [30,31] and the use of CAM-ICU should not replace clinical judgment [30]. In acute coronary syndrome, diagnosis by electrocardiogram is usually difficult but diagnosis by blood tests (e.g., detection of a combined increase in creatinine kinase and troponin T) is very easy and reliable in practice. The specificity of serum pNF-H detection for the diagnosis of delirium is at least similar to that of CAM-ICU. In addition, the estimation of serum pNF-H levels might provide an objective evaluation of the severity of delirium, on the basis of our previous finding because serum levels of pNF-H is linearly correlated with the clinically-validated delirium severity [8]. Alternative to pNF-H, the S-100 beta (S100β) protein and the neuron-specific enolase (NSE) were identified and characterized as candidate biochemical surrogate markers for brain damage [32]. Among these, to our knowledge, there are no studies which biomarker demonstrates more useful clinically to diagnose of and evaluate the severity of delirium. Since biological half-lives in serum NSE and S100β were shorter (approximately 48 h) than that of pNF-H (approximately 96h) [33], more critical time window would be required to diagnose of and evaluate of the severity of delirium by NSE and S100β than pNF-H. As one of candidate biomarkers, measuring serum pNF-H is expected to be a simple and objective delirium diagnostic assessment in clinical settings, and it may be superior to conventional clinical diagnostic tools, such as CAM-ICU which require implementation by experienced physicians [20].

There are three limitations in the present study. First, the screening of the patients was based on the clinical evaluation performed by the attending nurses. Therefore, we identified mainly hyperactive delirium cases. However, three clinical subtypes of delirium (hyperactive, hypoactive, and mixed) are known; hypoactive delirium is more frequent than hyperactive delirium and its clinical outcome is also poor [34,35]. To diagnose hypoactive and mixed delirium, we should create a diagnostic tool that could be applied on a 24-h basis by trained evaluators. Furthermore, future studies should include hypoactive and mixed delirium cases. Second, serum pNF-H was measured within 72 h after surgery in this study, on the basis of the previous notion that in spinal cord injury cases the most suitable time window for the measurement of serum pNF-H was between 18 and 96 h [21]. However, the most appropriate time for measuring serum pNF-H as an indicator of postoperative delirium-related CNS damage may be different from that in the case of spinal cord injury. Therefore, it is necessary to estimate the time course of serum pNF-H in postoperative patients. Third, serum pNF-H levels reportedly increased in some CNS disorders like as Alzheimer’s disease, the fronto-temporal dementia and fever convulsion [36,37]. Enrolling the participants, we excluded patients with clinically-relevant cognitive dysfunction before surgery. At the time of measuring serum pNF-H, we might not clearly discriminate postoperative delirium from clinical presentations of acute exacerbation of CNS disorders. These clinical presentations sometimes closely resemble each other, but in any case our present findings of elevating serum pNF-H levels can directly indicate CNS damage in the acute postoperative period.

In conclusion, to investigate the relationship among postoperative delirium, CNS damage, and BBB dysfunction we examined the levels of serum biomarkers of BBB disruption, pro-inflammatory cytokines, and pNF-H, in patients that underwent surgery. P-selectin was associated with serum pNF-H detection, indicating that it might be involved in the development of delirium-related CNS damage. PECAM-1 was associated with increased pNF-H levels, which could be considered as a means to evaluate the clinical severity of delirium-related CNS damage, in patients with serum pNF-H detection. Our findings suggest that P-selectin and PECAM-1 are two cell adhesion molecules of the BBB potentially participating in the development and progression of postoperative delirium-related CNS damage in reference to the axonal damage biomarker, serum pNF-H.

## Supporting information

S1 Table Minimal dataset. (XLSX)
